# Does stroke-associated pneumonia play an important role on risk of in-hospital mortality associated with severe stroke? A four-way decomposition analysis of a national cohort of stroke patients

**DOI:** 10.1177/17474930231177881

**Published:** 2023-06-06

**Authors:** Matthew Gittins, Marco Antonio Lobo Chaves, Andy Vail, Craig J Smith

**Affiliations:** 1 Centre for Biostatistics, The University of Manchester, Manchester, UK; 2 Manchester Centre for Clinical Neurosciences, Geoffrey Jefferson Brain Research Centre, Manchester Academic Health Science Centre, Salford Royal NHS Foundation Trust, Salford, UK; 3 Division of Cardiovascular Sciences, School of Medical Sciences, The University of Manchester, Manchester, UK; 4 GSK Biologicals, Wavre, Belgium

**Keywords:** SAP, epidemiology, risk factors, stroke, pneumonia, in-hospital mortality

## Abstract

**Background:**

Severe strokes and stroke-associated pneumonia (SAP) have long been associated with poorer patient health outcomes, for example, in-hospital mortality. However, it is unclear what role SAP plays in the risk of in-hospital mortality associated with a severe stroke at admission.

**Methods:**

Using the Sentinel Stroke National Audit Program data on stroke admissions (2013–2018) in England and Wales, we modeled the “total” effect for severe stroke on risk of in-hospital mortality. Through four-way decomposition methodology, we broke down the “total” observed risk into four components. The direct “severity on outcome only” effect, the pure indirect effect of severity mediated via SAP only, the interaction between severity and SAP when mediation is not present, and when mediation via SAP is present.

**Results:**

Of 339,139 stroke patients included, 9.4% had SAP and 15.6% died in hospital. Of SAP patients, 45% died versus 12% of non-SAP patients. The risk ratio for in-hospital mortality associated with severe versus mild/moderate stroke (i.e. total effect) was 4.72 (95% confidence interval: 4.60–4.85). Of this, 43%-increased risk was due to additive SAP interaction, this increased to 50% for “very severe” stroke. The remaining excess relative risk was due to the direct severity on outcome effect only, that is, there was no evidence here for a mediation effect via SAP.

**Conclusion:**

SAP was associated with a higher mortality in severe stroke patients. Prioritizing SAP prevention in severe stroke patients may improve in-hospital survival. Our results suggest that in severe stroke patients avoiding SAP might result in an up to 43% reduction in mortality.

## Introduction

As a leading cause of mortality and disability stroke is considered to have a significant burden on public health. In 2019, stroke accounted for approximately 11% or six million deaths worldwide, second only to ischaemic heart disease.^1^ One contributing factor is the occurrence of stroke-associated pneumonia (SAP). SAP is defined as pneumonia occurring within the first 7 days of a stroke patient’s admission to hospital. It has been estimated that SAP occurs in 8–13% of stroke patients.^2^ Previous work indicated that SAP is associated with older age, occurrence of dysarthria, dysphagia, cognitive impairment, and more severe stroke.^3^ Stroke severity has been repeatedly identified as a key predictive factor associated with SAP,^4,5^ with the relationships presented indicating an increased odds of SAP of between 3 and 16 for severe stroke patients (National Institute of Health Stroke Scale (NIHSS) > 15) compared to non-severe.^4^ In turn, patients with SAP are commonly accepted to be at increased risk of mortality,^3,6,7^ increased disability,^6^ and longer lengths of stay within hospital.^6,8^ Specifically, short-term mortality (within 3 months) and in-hospital mortality have been associated with SAP patients, with hazard ratio (HR) of 2.3 (95% confidence interval (CI): 2.1–2.5) and 2.4 (95% CI: 2.3–2.4), respectively.^6^

**Figure 1.**
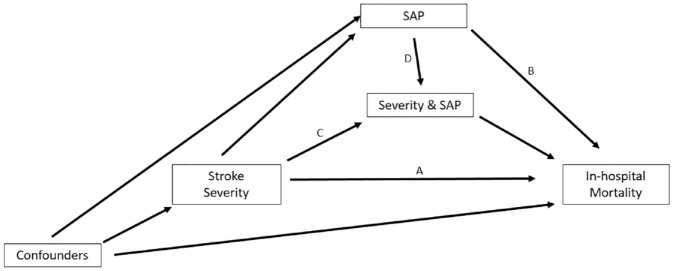
Proposed hypothetical causal relationships for the total effect of stroke severity on in-hospital mortality broken down into the direct effect (Route A), pure indirect effect via SAP (Route B), the reference severity by SAP additive interaction for SAP not due to severity (Route C), or mediated SAP additive interaction for the portion of SAP that was due to severity (Route D).

A key clinical question is whether preventing SAP in patients with a severe stroke could lead to improved health outcomes such as improved in-hospital survival. As SAP occurs after admission potentially as a result of having a severe stroke, it should not be considered as a confounder in any analysis investigating baseline characteristics such as stroke severity on admission. To do so would result in biased effect estimates of the baseline characteristics.^9^ Instead, SAP should be considered a patient characteristic that potentially contributes to the risk of in-hospital mortality either as a mediator, that is, severity may cause SAP which in turn causes in-hospital mortality, or as an interaction, that is, patients with severe stroke and SAP experience increased risk of mortality. Figure 1 depicts these proposed hypothetical causal relationships between severity, SAP, and in-hospital mortality. To explore post admission factors (i.e. SAP), studies may look for common associations between how severity indicators predict SAP and how severity indicators predict outcomes. Alternatively, SAP may also be evaluated in terms of health outcomes for those who survived post 7 days in hospital (i.e. patients were observed to have developed SAP or not). These methods provide useful insight into the relationships present. However, they do not adequately assess the role of SAP as a mediator in a complete population of patients who reach hospital.

Here, we aimed to better understand the role SAP plays within the chain of events (Figure 1) between admission to hospital with a severe stroke and in-hospital mortality. We employed causal mediation analysis techniques to break down the “total” (i.e. overall) effect of stroke severity on in-hospital mortality into component parts due to the presence or not of SAP. This allows us to explore the extent to which SAP contributes to risk of in-hospital mortality associated with stroke severity and address the following questions: Are severe patient’s high risk regardless of their SAP status? If not, how much mortality risk is contributed by SAP occurrence? Does stroke severity itself influence the presence of SAP which in turn influences mortality risk? How much of the “total” risk due to severity is due to this mediation via SAP. An ultimately, if we prevented SAP how much risk associated with a severe stroke could be removed?

## Methods

### Four-way decomposition model

To gain better insights into these causal relationships between stroke severity, SAP and in-hospital mortality, we employed four-way decomposition methodology as proposed by VanderWeele^10^ under the counterfactual approach to causal mediation analysis. Figure 1 uses a Directed Acyclic Graph (DAG) to illustrate our proposed hypothetical causal relationship between stroke severity and in-hospital mortality. The DAG attempts to graphically represent the additional presence of our mediator SAP, an interaction between severity and SAP, and a set of confounding factors, that is, secondary factors associated with stroke severity, SAP, and in-hospital mortality (factors such as age, stroke type, or comorbidities that are recorded at baseline admission).^11,12^ The total effect, that is, the overall effect of severity on in-hospital mortality is decomposed here into four components.

1. The effect of stroke severity on in-hospital mortality not through SAP (i.e. when the mediator SAP is fixed to no SAP), called the controlled direct effect (Figure 1, Route A).
2. The additive interaction effect of stroke severity and SAP alone (i.e. no mediation where SAP is present but not due to stroke severity), called the reference interaction effect (Figure 1, Route C).

Note, the pure direct effect is the sum of the controlled direct effect and the reference interaction, that is, the direct effect of stroke severity on in-hospital mortality not indirectly through SAP.

3. The additive interaction effect of stroke severity and SAP only when severity has an effect on SAP (i.e. when SAP is caused by a severe stroke), called the mediated interaction effect (Figure 1, Route D).
4. The effect of a severe stroke on in-hospital mortality through SAP (i.e. if SAP affects the outcome, even when stroke severity is absent, but stroke severity affects SAP presence), called the main mediated effect or the pure indirect effect (Figure 1, Route B).

Note, the natural indirect effect is the sum of the mediated interaction effect and pure indirect effect, that is, the indirect effect of stroke severity on in-hospital mortality due to severities effect on SAP.^13^

**Figure 2.**
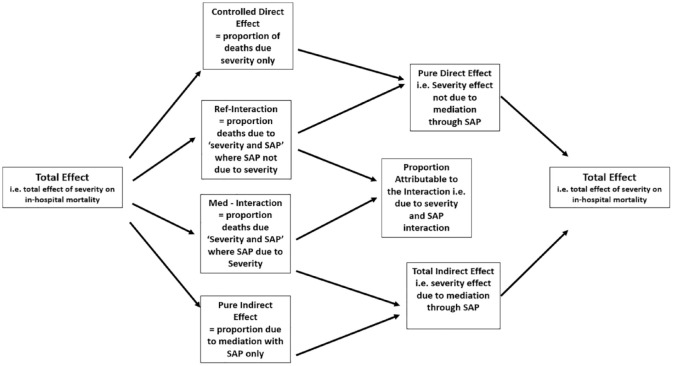
Breakdown of the total effect due to stroke severity, into the four component parts, (and their subsequent inter-relationships) relating to the role of SAP.

In addition to reporting the four components, the controlled direct effect, the reference interaction effect, the mediated interaction effect, and the pure indirect effect, we are also able to report the proportion of each component relative to the total excess relative risk (1 − the risk ratio) associated with stroke severity on in-hospital mortality. Furthermore, we are able to estimate the overall proportion attributable to the interaction (reference and mediated combined), the overall proportion mediated (pure indirect plus mediated interaction), and the proportion of the effect that would be eliminated if the mediator (SAP) was prevented from occurring, that is, the excess relative risk minus the controlled direct effect.^14^ This indicates the amount of excess relative risk that could be prevented if SAP could be prevented. Figure 2 describes these relationships.

### Setting

Patient level data on all stroke admissions in England and Wales between 1 April 2013 and the 31 December 2018 were requested from the Sentinel Stroke National Audit Program (SSNAP).^15^ Patients with ischaemic or haemorrhagic stroke were included, though patients at stroke units with less than a maximum of 150 patients in any of the 5-year period were excluded.^16^ This was to better reflect patients experience at established specialist stroke units. Patients with a time from stroke onset to hospital admission greater than 24 h were also excluded, to avoid delayed stroke presentations where pneumonia may already have developed between stroke onset and admission.

### Stroke severity

Stroke severity was defined using the NIHSS recorded on admission.^17^ Missing data were present in components of the NIHSS, except level of consciousness (LOC). To account for this missing data, we used LOC (0 = 0, 1 = 5, 2 = 15, and 3 = 20) + non-LOC NIHSS total score to define NIHSS severity categorized as 0–4 = mild, 5–15 = moderate, 16–20 = severe, and >21 = very severe.

### SAP

SSNAP defines SAP as present if the patient was recorded as having a new antibiotic initiation for pneumonia within the first 7 days of stroke admission. This can occur in SSNAP at any point within the first 7 days regardless of patient discharge or death in hospital.

### Primary outcome—in-hospital mortality

SSNAP only records information on patients during their time in hospital, this includes time to inpatient discharge and reason for discharge. The primary outcome was therefore time in days from hospital admission to discharge due to death.

### Sources of confounding

Any association between severe stroke, SAP, and in-hospital mortality will be confounded by secondary characteristics of the participant population recorded at the hospital admission. Sources of confounding were identified from the SSNAP data to be: sex, age grouped, ethnicity, pre-stroke disability (modified Rankin scale (mRs)), stroke type (not including subarachnoid hemorrhage), previous stroke, comorbidities chronic heart failure, hypertension, atrial fibrillation, and diabetes, and the size of a stroke unit (maximum number of patients per year). Patient socioeconomic status based on indices of multiple deprivation (IMD) of the hospital trust. Annual trends in stroke characteristics or care, seasonal changes in pneumonia prevalence or co-infection, and day of the week changes to stroke severity and patient care were all accounted for. Finally, dysphagia defined as patients with a baseline swallow screen followed either by an Speech and Language Therapy (SLT) assessment or no SLT assessment due to being “too unwell” or for organizational reasons.^18^

### Statistical analysis

Statistical analysis was performed using Stata, version 17, and the med4way command to obtain appropriate estimates of the four components.^19,20^ A logistic regression model was fitted to SAP presence and a Cox proportional hazards model to the time to in-hospital death. The logistic model included stroke severity adjusted for covariates and clustering by stroke unit. The Cox model included an interaction term between stroke severity and SAP and accounted for stroke unit using a shared frailty model. The censor date was their last known alive date, that is, on discharge. The analysis was repeated under three definitions of severity. First, “severe/very severe” (severe+) stroke versus “mild/moderate” and “very severe” versus “mild/moderate/severe” stroke. An third in a sensitivity analysis of our definition of severity using LOC score ⩾2 on admission which will also help understand if the LOC may be a suitable surrogate. Here, appropriate parameter estimates to calculate the four components of the decomposition model were obtained using the regression based approach outlined by VanderWeele.^10^ This included converting the multiplicative interactions modeled on a ratio scale to an approximate additive interaction by calculating the Relative Excess Risk due to Interaction (RERI).^10,21^ The estimates of the four components are presented, and standard errors and CIs were obtained using the delta method.^10^

## Results

The initial data provided by SSNAP included 458,829 stroke patients. Inclusion criteria meant 2378 patients were dropped due to the time to hospital discharge/death in hospital being less than zero. There were 141 stroke units, representing 22,337 patients, which had less than a maximum of 150 patients in all years (2013–2018). A further 94,975 patients were dropped due to time between onset to hospital admission being greater than 24 h. Of the 339,139 stroke patients remaining 9.4% were identified to have SAP. Overall, 15.7% of the sample died within hospital, 45.8% of those with SAP. Median time to death was 7 days (interquartile range (IQR): 2.7–19.5) from admission, 99% of all deaths occurred within 108 days. Of the 339,139 patients in the study, 20,144 were discharged to palliative care. However, of those only 2227 (0.66% of the sample) were not also identified in our data as having died in hospital. Approximately 8% of patients had missing data in each of the NIHSS components. Within LOC groups, the median NIHSS for those with an LOC score of 2 and 3 were 21 and 24 with missing data but 23 and 30 when missing data were excluded (see Supplementary Tables 2 and 3). Those patients identified as severe and very severe were at increased probability of SAP, 19.5% and 23.2%, respectively, compared to moderate (8.8%) and mild (3.5%) severity. In addition, 30% of severe and 54% of very severe patients died in hospital. Full descriptive statistics can be found in Table 1 and in Supplementary Table 1.

**Table 1.** Descriptive frequency and percentage of baseline covariates by SAP, and in-hospital mortality for those entering hospital within 1 day.

Covariate	Factor	SAP (%)	Died (%)	Total
SAP status	Non-SAP	—	38,582 (12.6)	307,156
	SAP	—	14,662 (45.8)	31,983
NIHSS severity	Mild (<5)	5449 (3.5)	6049 (3.9)	154,046
	Moderate (5–15)	9493 (8.8)	11,466 (10.7)	107,573
	Severe (15–20)	4971 (19.5)	7627 (29.9)	25,526
	Very severe (>20)	12,070 (23.2)	28,102 (54.0)	51,994
Level of consciousness	⩽2	26,828 (8.5)	37,267 (11.8)	315,046
	>2	5155 (21.4)	15,977 (66.3)	24,093
Sex	Female	16,486 (9.8)	30,861 (18.3)	168,750
	Male	15,497 (9.1)	22,383 (13.1)	170,389
Ethnicity	White	707 (7.7)	998 (10.8)	9223
	Asian	226 (6.0)	368 (9.8)	3771
	Black	99 (9.1)	131 (12.1)	1084
	Mixed	1480 (8.6)	2489 (14.5)	17,215
	N/A	333 (9.3)	401 (11.2)	3575
	Other	28,477 (9.6)	47,934 (16.1)	297,604
Age grouped	<60	1639 (3.5)	1936 (4.2)	46,177
	60–69	3037 (5.8)	4039 (7.7)	52,589
	70–79	7758 (8.6)	11,337 (12.6)	90,133
	80–89	13,367 (12.2)	23,175 (21.1)	109,575
	>90	6182 (15.2)	12,757 (31.4)	40,665
Comorbidities	Congestive heart failure	2815 (14.7)	4970 (26.0)	19,141
	Hypertension	17,616 (9.6)	28,558 (15.6)	182,580
	Atrial fibrillation	9746 (13.6)	17,745 (24.8)	71,462
	Diabetes	6717 (9.8)	10,692 (15.6)	68,542
	Previous stroke or TIA	9229 (9.9)	15,330 (16.5)	92,856
Stroke subtype	Ischaemic stroke	27,032 (9.0)	39,268 (13.1)	299,028
	Primary ICH	4951 (12.3)	13,776 (34.3)	40,111
	Dysphagia	31,050 (9.5)	52,079 (16.0)	325,919
Pre-morbid modified Rankin scale	0	12,074 (6.6)	17,863 (9.8)	181,778
	1	4909 (9.6)	7661 (14.9)	51,278
	2	4324 (12.2)	7247 (20.5)	35,360
	3	5958 (14.4)	11,108 (26.9)	41,350
	4	3626 (16.0)	7099 (31.3)	22,666
	5	1092 (16.3)	2266 (33.8)	6707
Total		31,983 (9.4)	53,244 (15.7)	339,139

SAP: stroke-associated pneumonia; NIHSS: National Institute of Health Stroke Scale; NA: not applicable; TIA: transient ischemic attack; ICH: intracranial hemorrhage.

**Figure 3.**
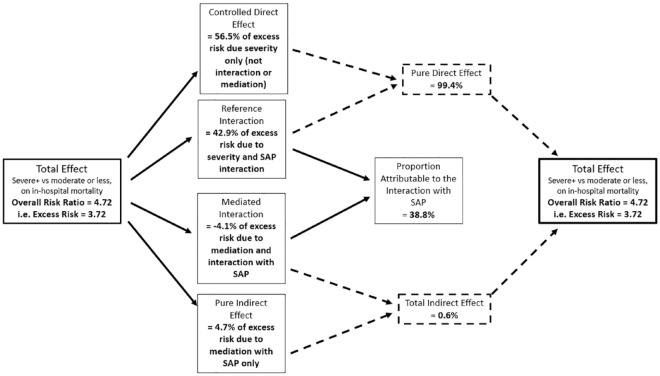
Breakdown of the total effect experience by severe stroke patients (NIHSS </> 15) in to its four component parts due to mediation through SAP and additive interaction with SAP; dashed lines represent pure direct and total indirect effects which sum to the total effect.

Table 2 reports the “total effect” relative risk associated with patients NIHSS severe or worse stroke, that is, “severe+” versus not and separately as a “very severe” stroke versus not. The “total effect” risk ratio for severity in both models was similar at 4.72 (4.60–4.85) and 4.73 (4.60–4.86), respectively. This corresponds to an excess relative risk (the additional risk due to severe stroke) of 3.72 and 3.73, respectively. Table 2, Figure 3, and Supplementary Figure 1 (for very severe) illustrate how the “Total” excess relative risk for stroke severity has been broken down into the component parts. The first half of the table reports the effect estimates as produced by the regression model and subsequent decomposition. The second half describes in terms of the proportion of “Total” excess risk due to stroke severity accounted for by the component parts. The controlled direct effect (i.e. the effect of stroke severity alone) accounts for the largest proportion of the excess relative risk at 56.5% for the severe+ versus not and 50.7% for very severe versus not. The majority of the remaining excess relative risk is accounted for by the additive interaction only (i.e. not due to mediation through SAP) between stroke severity and SAP, with 43% and 50% of the excess relative risk, respectively. The influence of SAP as a mediator appears to be negligible, with the proportion of excess relative risk due to both interaction and mediation via SAP is −4.1% and −4.0% and due to mediation only (the pure indirect effect) is 4.7% and 3.3%, respectively. The proportion of overall excess relative risk due to mediation was 0.6% and −0.7%, whereas the overall proportion of excess relative risk due to the additive interaction of stroke severity and SAP was 38.8% and 45.5%, respectively. The proportion eliminated, that is, the total proportion of the risk that could be removed if SAP was prevented from occurring is 43.5% and 49.5%, respectively, for severe and very severe stroke. The results of the additional LOC analyses can be found in Supplementary Table 4 and Supplementary Figure 2. Using NIHSS LOC (⩾2 vs <2) as an alternative for patient severity, the “Total effect” risk ratio was 5.42 (5.26–5.58). The portion of the excess relative risk due to the controlled direct effect was 37.6%, and the overall interaction effect increased to 61.6%. This resulted in a total proportion eliminated if SAP could be prevented from occurring in these patients of 62.4%.

**Table 2.** Results of the four-way decomposition model investigating the role of SAP (mediator) on the association between stroke severity and within hospital mortality in those admitted within 1 day of stroke onset (N = 339,139).

Effect estimates of interest (95% CI) ABCD = path in Figure 1	NIHSS	NIHSS
	Severe+ versus mild/moderate	Very severe versus mild/moderate/severe
Total effect risk ratio—1 + A + B + C + D	4.72 (4.60 to 4.85)	4.73 (4.60 to 4.86)
Total excess relative risk—A + B + C + D	3.72 (3.60 to 3.85)	3.73 (3.60 to 3.86)
Total excess relative risk broken down into components		
Controlled direct effect = A	2.10 (1.96 to 2.24)	1.89 (1.76 to 2.02)
Interaction (reference) = C	1.60 (1.45 to 1.74)	1.86 (1.72 to 2.01)
Interaction (mediated) = D	−0.15 (−0.19 to −0.11)	−0.15 (−0.19 to −0.11)
Pure indirect effect = B	0.18 (0.13 to 0.22)	0.12 (0.09 to 0.15)
Proportion of total excess relative risk per components		
Controlled direct effect = A	56.5 (53.1 to 59.8)	50.7 (47.6 to 53.8)
Interaction (reference) = C	42.9 (39.3 to 46.5)	50.0 (46.7 to 53.4)
Interaction (mediated) = D	−4.1 (−5.2 to −3.0)	−4.0 (−5.2 to −2.9)
Pure indirect effect = B	4.7 (3.4 to 6.0)	3.3 (2.4 to 4.2)
Combined proportions of excess relative risk		
Due to mediation = B + D	0.6 (0.3 to 0.9)	−0.7 (−1.1 to −0.4)
Due to interaction = C + D	38.8 (35.3 to 42.3)	46.0 (42.7 to 49.3)
Eliminated if SAP prevented = B + C + D	43.5 (40.2 to 46.9)	49.3 (46.2 to 52.4)

SAP: stroke-associated pneumonia; CI: confidence interval; NIHSS: National Institute of Health Stroke Scale.

## Discussion

The results presented here provide further evidence for the importance of preventing SAP in patients. The risk of in-hospital mortality experienced by severe stroke patients may be reduced if SAP can be prevented. Here, we observed 39% of the excess relative risk of in-hospital mortality experienced by severe stroke patients was due to the additive interaction between stroke severity and SAP, this increased to 46% in very severe patients and 61% in those with severe LOC. In other words, patients with both a severe stroke and SAP together had ≈40% increased risk of in-hospital mortality to those with just a severe stroke or just SAP. This increases to 60% in patients whose LOC is considered severe. Therefore, reducing the occurrence of SAP could likely result in a disproportionate improvement in survival of severe stroke patients. Interestingly, SAP does not appear to be behaving as a mediator, that is, with respect to risk of mortality in hospital all of the severe stroke risk was direct (controlled direct effect plus reference interaction effect 99.4%) and not acting indirectly through SAP (0.4%). This suggests the influence of stroke severity on in-hospital mortality was not through increasing the risk of SAP.

Our results suggest that at least 43.5% (proportion eliminated) of excess relative risk of mortality associated with stroke severity would be removed if we could prevent SAP. With around 40% of the excess relative risk being attributable to patients having both a severe stroke and SAP compared with only one of each, our findings indicate that understanding the mechanisms, identifying risk factors, and developing preventive measures to reduce the occurrence of SAP are important, particularly in severe stroke patients. Note, due to the explicit assumptions of the study methodology, the possible reduction in risk would not occur if SAP was treated, only prevented in the first place. Preventing SAP is a major challenge for all stroke units, particularly in very severe patients. Recent clinical trials have attempted to reduce the occurrence of SAP and in turn improve health outcomes. The Preventive Antibiotics in Stroke Study (PASS)^22^ and STROKE-INF^23^ aimed to reduce the occurrence of SAP and improve subsequent outcomes using preventive antibiotic treatment in either unselected patients or in those patients with dysphagia after acute stroke, respectively. Results from the PASS study indicated that preventive antibiotics did not show strong evidence of a reduction in new cases of pneumonia during hospital admission. These studies were included as part of a wider individual patient data meta-analysis of preventive antibiotics which indicated no overall associated improvement in mRs or short-term mortality due to preventive antibiotic treatment.^24^

Why preventive antibiotic treatment was unable to prevent pneumonia after stroke is unclear,^25^ but the results of our study add to the need for further work to better understand the mechanisms and pathophysiology of SAP in order to improve prediction algorithms, diagnostic accuracy, understanding of current treatment, and development of new treatments and preventive strategies.^25,26^ Specifically, improvements are required in defining the underlying pathogens associated with SAP, and the subsequent optimal treatment options.^27,28^ In addition, there are currently no agreed upon guidelines for the diagnosis, prevention, and management of SAP.^26^ This would be key to any improvements in the prospects of severe stroke patients with pneumonia specifically with the development of any preventive interventions, treatment, and understanding of key risk factors.

### Limitations/assumptions

The SSNAP data employed here were observational in nature, were based on clinical records collected as part of daily routine and not as part of a prospective study, and were more focused on care processes. This results in significant advantages and disadvantages for our study. The sample size was large, covers a recent period of time, and was representative of a significant proportion (>95%) of the stroke admissions occurring within England and Wales. The records-based nature of the data means that it may be considered representative of true experience of all stroke patients and their care. It also includes longitudinal follow-up of key pieces of data, for example, antibiotics prescription within 7 days post admission, and in-hospital mortality follow-up. This allows for us to be confident that any observed relationship was temporal in nature. We are however reliant on the accuracy of the data available, the level of missing data, and the possibility of misclassification. We have relied on prescription of antibiotics within 7 days for pneumonia to define the presence of SAP, although there may be some misclassification present if patients are not being treated with antibiotics despite having pneumonia due to the severity of their condition and perceived futility of treatment. Missing data were present within the NIHSS components used to define severity, particularly within the severely unconscious who have an LOC score of 3 (i.e. very serve), but a low total NIHSS score (so apparent mild stroke). We accounted for this by using the LOC score and the NIHSS score in combination to define a bespoke categorical variable of severity. This may have resulted in misclassification especially if missing NIHSS components were not missing completely at random. Our adjustment may cause some milder patients to have their severity overestimated underestimating the risks associated with severity on outcomes. However, we would expect the risks to experience greater underestimation if the adjustment for missing NIHSS components was not performed due to severe and very severe patients being misclassified as mild. This may be reflected in the results when LOC itself was used as a proxy. We were also limited to in-hospital mortality, meaning anyone seeking end of life care at home or in a care home, or anyone deemed fit enough for discharge from hospital but who subsequently deteriorated possibly due to SAP and died would not have been appropriately included. With respect to end of life care, we observed 2227 (0.7% of all patients) requiring palliative care but did not register as an in-hospital mortality; however, it is unclear if others may have been misclassified. It is also worth noting, that the analysis here is reliant on the rare-event assumption, making estimates of risk for mortality comparable. This means we were only able to focus on mortality and could not justify expanding this analysis to other characteristics such as severity, disability, or length of stay. This means we can make no conclusions about the characteristics of severe stroke patients who would benefit from preventing SAP.

As a stand-alone analysis, the four-way decomposition model is considered to provide valid results; however, when free of bias under a set of strong assumptions the results can provide important insight into causal relationships between stroke severity, SAP, and in-hospital mortality. VanderWeele highlighted the following assumptions concerning confounding conditional on the baseline set of covariates^10,14^:

1. The effect of stroke severity on in-hospital mortality should not be confounded (important for the total effect).
2. The effect of SAP on incident of in-hospital mortality should not be confounded, conditional on stroke severity (important in addition to the previous assumption for the controlled direct effect).
3. The effect of stroke severity on SAP should not be confounded.
4. The mediator-outcome confounders should not be affected by stroke severity.

To ensure these assumptions hold, we have made every effort to account for an appropriate set of confounders, including unmeasured confounders associated with the stroke unit. However, we were limited by the information recorded and available in SSNAP and cannot be sure we have accounted for all important confounders.

## Conclusion

Our findings have suggested that preventing SAP in severe stroke patients should be a key priority in the management of stroke patients on admission. Through improved preventive measures focusing on reducing the occurrence of SAP in severe patients we could reduce the risk of in-hospital mortality experienced by 40%, and greater in very severe stroke patients. Interestingly SAP is having minimal influence as a mediator, in other words the effect of severity on in-hospital mortality does not appear to be due to its effect on increasing the risk of SAP. However, SAP appears to play an important role on in-hospital mortality risk through its interaction with stroke severity. Those patients who have severe stroke and SAP are at substantial increased risk compared to those with severe stroke or SAP only.

## Supplemental Material

sj-docx-1-wso-10.1177_17474930231177881 – Supplemental material for Does stroke-associated pneumonia play an important role on risk of in-hospital mortality associated with severe stroke? A four-way decomposition analysis of a national cohort of stroke patients Click here for additional data file. Supplemental material, sj-docx-1-wso-10.1177_17474930231177881 for Does stroke-associated pneumonia play an important role on risk of in-hospital mortality associated with severe stroke? A four-way decomposition analysis of a national cohort of stroke patients by Matthew Gittins, Marco Antonio Lobo Chaves, Andy Vail and Craig J Smith in International Journal of Stroke
