# Supplementary material for: Does stroke-associated pneumonia play an important role on risk of in-hospital mortality associated with severe stroke? A four-way decomposition analysis of a national cohort of stroke patients
Source: Int J Stroke. 2023 Jun 6;18(9):1092–101. doi: 10.1177/17474930231177881 (PMC10614175; doi:10.1177/17474930231177881)
Supplement: sj-docx-1-wso-10.1177_17474930231177881 – Supplemental material for Does stroke-associated pneumonia play an important role on risk of in-hospital mortality associated with severe stroke? A four-way decomposition analysis of a national cohort of stroke patients [file sj-docx-1-wso-10.1177_17474930231177881.docx]

**Supplementary Material**

**Supplementary Table 1** – Descriptive frequency and percentage of baseline covariates by SAP, and in-hospital mortality for those entering hospital within one day.

| **Covariate** | **Factor** | **No. SAP(%)** | **No. Died(%)** | **Total** |
| --- | --- | --- | --- | --- |
| Socio-economic Decile | 0 | 1486(8.8) | 2767(16.3) | 16956 |
|  | 1 | 2759(9.2) | 4312(14.4) | 29931 |
|  | 2 | 2673(9.2) | 4092(14.1) | 28935 |
|  | 3 | 2898(9.7) | 4580(15.4) | 29799 |
|  | 4 | 2901(9.4) | 4742(15.3) | 31005 |
|  | 5 | 3113(9.6) | 5266(16.2) | 32596 |
|  | 6 | 3150(9.5) | 5372(16.3) | 33000 |
|  | 7 | 3138(9.6) | 5387(16.4) | 32778 |
|  | 8 | 2988(9.5) | 5179(16.4) | 31603 |
|  | 9 | 2903(9.5) | 4990(16.3) | 30682 |
|  | 10 | 2726(9.5) | 4799(16.7) | 28707 |
|  | Miss | 1248(9.5) | 1758(13.4) | 13147 |
| Year of admission | 2013 | 3680(9.6) | 5807(15.2) | 38287 |
|  | 2014 | 5874(9.8) | 9442(15.8) | 59682 |
|  | 2015 | 5661(9.2) | 9938(16.2) | 61372 |
|  | 2016 | 6006(9.7) | 9875(16.0) | 61878 |
|  | 2017 | 5939(9.5) | 9809(15.6) | 62741 |
|  | 2018 | 4823(8.7) | 8373(15.2) | 55179 |
| Day of Week of Admission | Monday | 4793(9.5) | 7978(15.8) | 50635 |
|  | Tuesday | 4641(9.3) | 7787(15.5) | 50089 |
|  | Wednesday | 4713(9.4) | 7918(15.7) | 50303 |
|  | Thursday | 4628(9.4) | 7690(15.6) | 49405 |
|  | Friday | 4555(9.4) | 7430(15.3) | 48713 |
|  | Saturday | 4340(9.7) | 7158(15.9) | 44924 |
|  | Sunday | 4313(9.6) | 7283(16.2) | 45070 |
| Quartile of year of Admission | Q1 (Jan-Mar) | 7966(9.1) | 13265(15.1) | 87589 |
|  | Q2 (Apr-Jun) | 7879(9.0) | 12976(14.8) | 87611 |
|  | Q3 (Jul-Sep) | 8356(9.6) | 14277(16.5) | 86720 |
|  | Q4 (Oct-Dec) | 7782(10.1) | 12726(16.5) | 77219 |
| Stroke Unit Size (patient per year) | <300 | 3782(11.6) | 4788(14.7) | 32558 |
|  | 300-450 | 6816(10.5) | 10562(16.3) | 64693 |
|  | 450-600 | 7574(9.1) | 13608(16.4) | 82920 |
|  | 600-800 | 7385(8.9) | 12607(15.3) | 82611 |
|  | >800 | 6426(8.4) | 11679(15.3) | 76357 |
| Total |  | 31983(9.4) | 53244(15.7) | 339139 |

**Supplementary Table 2** – Descriptive statistics for NIHSS components including missing data (N=339,139).

| **NIHSS Component (All Stroke Patients 2013-18** | **0** | **1** | **2** | **3** | **4** | **Missing** | **Total** |
| --- | --- | --- | --- | --- | --- | --- | --- |
| Level Of Consciousness | 384980(83.9) | 43265(9.4) | 18319(4.0) | 12265(2.7) | - | - | 458,829 |
| LOC Questions | 276587(60.3) | 40054(8.7) | 101363(22.1) | - | - | 40825(8.9) | 458,829 |
| LOC Commands | 334124(72.8) | 33033(7.2) | 51379(11.2) | - | - | 40293(8.8) | 458,829 |
| Best Gaze | 335347(73.1) | 45821(10.0) | 33048(7.2) | - | - | 44613(9.7) | 458,829 |
| Visual | 300498(65.5) | 44198(9.6) | 56856(12.4) | 8121(1.8) | - | 49156(10.7) | 458,829 |
| Facial Palsy | 207809(45.3) | 130077(28.4) | 68843(15) | 11633(2.5) | - | 40467(8.8) | 458,829 |
| Motor Arm Left | 277179(60.4) | 61883(13.5) | 26961(5.9) | 16603(3.6) | 36128(7.9) | 40075(8.7) | 458,829 |
| Motor Arm Right | 287815(62.7) | 55266(12.1) | 23337(5.1) | 16417(3.6) | 35929(7.8) | 40065(8.7) | 458,829 |
| Motor Leg Left | 278883(60.8) | 52777(11.5) | 33205(7.2) | 23485(5.1) | 29652(6.5) | 40827(8.9) | 458,829 |
| Motor Leg Right | 289688(63.1) | 45502(9.92) | 29221(6.4) | 22663(4.9) | 30883(6.7) | 40872(8.9) | 458,829 |
| Limb Ataxia | 324536(70.7) | 56537(12.3) | 25894(5.6) | - | - | 51862(11.3) | 458,829 |
| Sensory | 277008(60.4) | 88853(19.4) | 43938(9.6) | - | - | 49030(10.7) | 458,829 |
| Best Language | 261752(57.1) | 62652(13.7) | 50752(11.1) | 42187(9.2) | - | 41486(9.0) | 458,829 |
| Dysarthria | 215995(47.1) | 134195(29.3) | 64206(14.0) | - | - | 44433(9.7) | 458,829 |
| Extinction Inattention | 305060(66.5) | 57373(12.5) | 50481(11) | - | - | 45915(10.0) | 458,829 |
| **NIHSS Component (Stroke Patients 2013-18 admitted within 1 day)** | **0** | **1** | **2** | **3** | **4** | **Missing** | **Total** |
| Level Of Consciousness | 279948(82.5) | 35098(10.3) | 14830(4.4) | 9263(2.7) | - | - | 339139 |
| LOC Questions | 198229(58.5) | 31083(9.2) | 82436(24.3) | - | - | 27391(8.1) | 339,139 |
| LOC Commands | 243595(71.8) | 26679(7.9) | 41909(12.4) | - | - | 26956(7.9) | 339,139 |
| Best Gaze | 244141(72) | 37000(10.9) | 27606(8.1) | - | - | 30392(9) | 339,139 |
| Visual | 220693(65.1) | 33342(9.8) | 44684(13.2) | 6249(1.8) | - | 34171(10.1) | 339,139 |
| Facial Palsy | 144158(42.5) | 101708(30) | 56655(16.7) | 9551(2.8) | - | 27067(8) | 339,139 |
| Motor Arm Left | 202103(59.6) | 45924(13.5) | 21249(6.3) | 13493(4) | 29560(8.7) | 26810(7.9) | 339,139 |
| Motor Arm Right | 210104(62) | 41079(12.1) | 18334(5.4) | 13438(4) | 29359(8.7) | 26825(7.9) | 339,139 |
| Motor Leg Left | 202805(59.8) | 39256(11.6) | 26283(7.7) | 19201(5.7) | 24174(7.1) | 27420(8.1) | 339,139 |
| Motor Leg Right | 211038(62.2) | 33891(10) | 23105(6.8) | 18470(5.4) | 25155(7.4) | 27480(8.1) | 339,139 |
| Limb Ataxia | 242082(71.4) | 41495(12.2) | 19258(5.7) | - | - | 36304(10.7) | 339,139 |
| Sensory | 200652(59.2) | 68282(20.1) | 36110(10.6) | - | - | 34095(10.1) | 339,139 |
| Best Language | 186451(55) | 48326(14.2) | 41603(12.3) | 34860(10.3) | - | 27899(8.2) | 339,139 |
| Dysarthria | 151536(44.7) | 104192(30.7) | 53097(15.7) | - | - | 30314(8.9) | 339,139 |
| Extinction Inattention | 219758(64.8) | 46136(13.6) | 41855(12.3) | - | - | 31390(9.3) | 339,139 |

**Supplementary Table 3** – Descriptive statistics for Total NIHSS score repeated to including missing data and with complete components for those admitted within 1 day.

| **NIHSS Total Score Missing Data Included** | **Level of Consciousness** | **N** | **Mean** | **SD** | **25th Percentile** | **Median** | **75th Percentile** |
| --- | --- | --- | --- | --- | --- | --- | --- |
| Missing NIHSS components Included | LOC=0 | 279948 | 5.77 | 5.74 | 2 | 4 | 8 |
|  | LOC=1 | 35098 | 15.54 | 7.61 | 10 | 16 | 21 |
|  | LOC=2 | 14830 | 19.37 | 9.08 | 14 | 21 | 26 |
|  | LOC=3 | 9263 | 20.42 | 13.38 | 3 | 24 | 31 |
|  | Total | 339139 | 7.78 | 7.84 | 2 | 5 | 12 |
| Complete NIHSS components Only | LOC=0 | 249978 | 6.03 | 5.64 | 2 | 4 | 8 |
|  | LOC=1 | 28330 | 16.50 | 7.00 | 11 | 17 | 22 |
|  | LOC=2 | 10403 | 22.14 | 7.10 | 18 | 23 | 27 |
|  | LOC=3 | 4915 | 28.86 | 8.91 | 24 | 30 | 35 |
|  | Total | 293626 | 8.00 | 7.72 | 2 | 5 | 12 |

**Supplementary Table 4** – Results of the four-way decomposition model investigating the role of SAP (mediator) on the association between stroke severity as LOC and within hospital mortality in those admitted within 1 day of stroke onset (N=339,139)

| **Effect estimates of interest** (95% C.I.)  ABCD = Path in Figure 1 | | **LOC** |
| --- | --- | --- |
|  |  | **≥2 v <2** |
|  |  |  |
| Total Effect Risk Ratio = 1+A+B+C+D |  | 5.42(5.26,5.58) |
| Total Excess relative risk = A+B+C+D |  | 4.42(4.26,4.58) |
| Total Excess Relative Risk broken down into components | Controlled Direct Effect = A | 1.66(1.52,1.81) |
|  | Interaction (Reference) = C | 2.82(2.64,3.01) |
|  | Interaction (Mediated) = D | -0.12(-0.15,-0.08) |
|  | Pure Indirect Effect = B | 0.05(0.04,0.07) |
| Proportion of total Excess Relative risk per components | Controlled Direct Effect = A | 37.6(34.4,40.8) |
|  | Interaction (Reference) = C | 63.8(60.5,67.2) |
|  | Interaction (Mediated) = D | -2.6(-3.4,-1.8) |
|  | Pure Indirect Effect = B | 1.2(0.8,1.5) |
| Combined Proportions of Excess relative risk | Due to Mediation = B+D | -1.4(-1.9,-1.0) |
|  | Due to Interaction = C+D | 61.2(58.0,64.5) |
|  | Eliminated if SAP removed = B+C+D | 62.4(59.2,65.6) |

LOC = NIHSS Level of Consciousness measure.

**Supplementary Figure 1 –** Breakdown of the total effect experience by very severe stroke patients (NIHSS </≥21) on in-hospital mortality in to its four component parts due to mediation through SAP and additive interaction with SAP; Dashed lines represent Pure Direct and Total Indirect Effects which sum to the Total Effect..


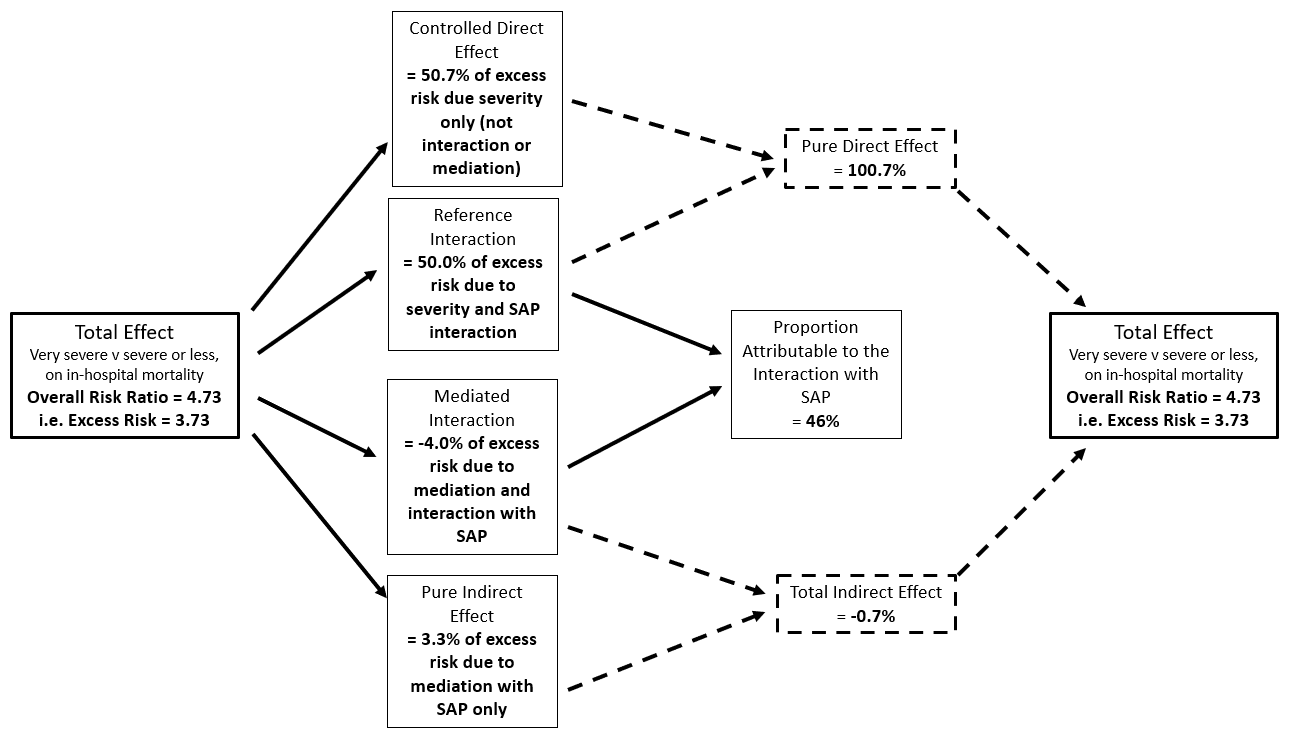


**Supplementary Figure 2 –** Breakdown of the total effect experienced by severe Loss of Consciousness in stroke patients (NIHSS LOC </≥2) on in-hospital mortality, in to its four component parts due to mediation through SAP and additive interaction with SAP; Dashed lines represent Pure Direct and Total Indirect Effects which sum to the Total Effect..


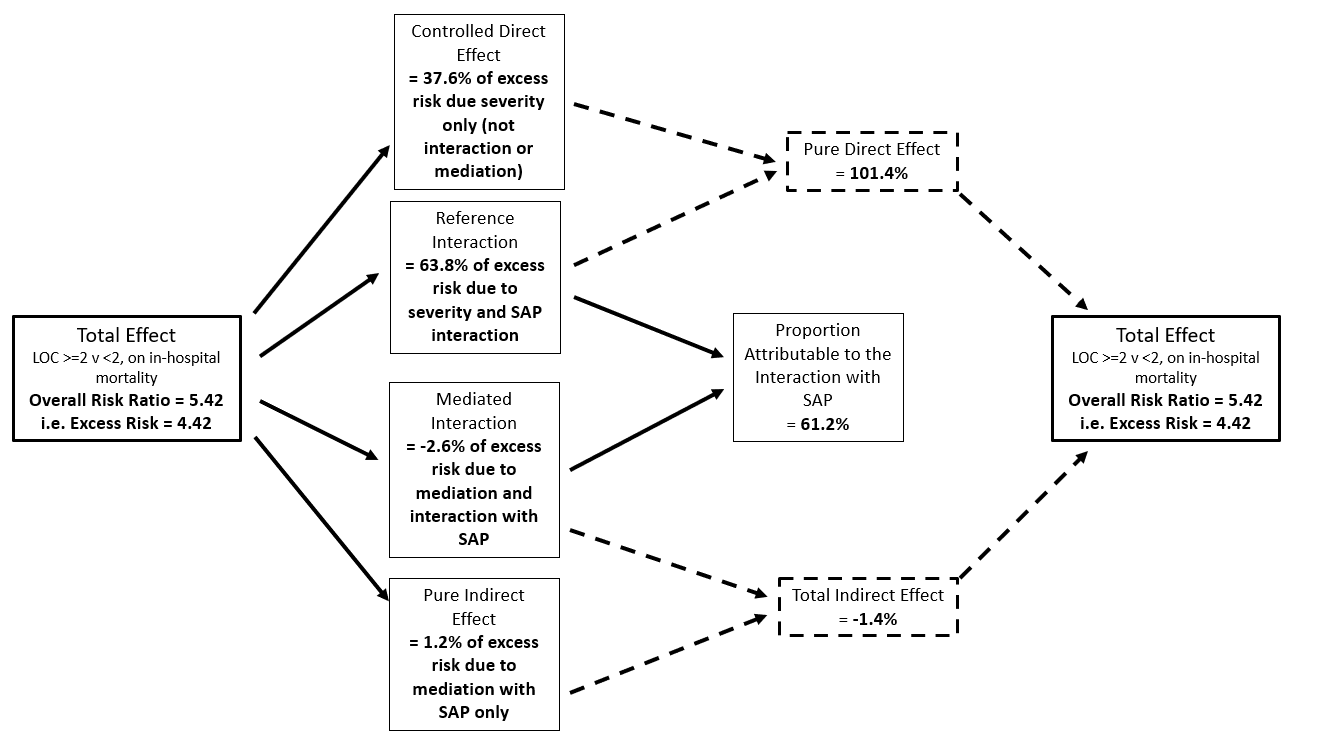


**Example Stata Code used to for the Analysis**

**Paper and Installation of ‘med4way’ (links as of Mar 2023)**

*Med4way paper =* [*https://pubmed.ncbi.nlm.nih.gov/30452641/*](https://pubmed.ncbi.nlm.nih.gov/30452641/)

*Github code =* [*https://github.com/anddis/med4way*](https://github.com/anddis/med4way)

Step 1 - Installed ‘med4way’ using

- net install med4way, from("https://raw.githubusercontent.com/anddis/med4way/master/") replace

Step 2 - Prepare data for analysis

- Generate indicator of event (e.g. in this analysis in-hospital death Yes/No)
- Generate time to event including time to censor date
- Generate indicator of mediator (i.e. SAP presence binary Yes/No)
- Generate indicator of ‘exposure’ (i.e. severity presence binary Severe/not)
- Generate/define set of model covariates representing confounders (e.g. gender, age, etc)

Step 3 – Use stset to define the survival data in STATA

For example

stset timetoevent, failure(death) noshow

Step 4 - Using ‘med4way’ command fit regression models for 4 way decomposition model

For example

xi: med4way nihss_severe sap7days i.gender_n i.ethnicity_n i.agerange_n i.decile i.s2rankinbeforestroke i.admissionyear_c i.admissionday_n i.admissionquarter_n i.su_size

i.chf_n i.htn_n i.afib_n i.diabetes_n i.stroketype_n i.prevstroketia_n i.dysphagia_n i.onsetinhospital_n i.onsetarrival_dys_cat , a0(0) a1(1) m(1) yreg(cox) mreg(logistic) mregoptions(cluster(teamcode) vce(cluster teamcode)) yregoptions(shared(teamcode)) c(1 0 0 0 0 0 0 0 0 0 0 0 0 0 0 0 0 0 0 0 0 0 0 0 0 0 0 0 0 0 0 0 0 0 0 0 0 0 0 0 0 0 0 0 0 0 0 0 0 0 0 0 0 0 0 0) full

Where

- ‘exposure’ variable = nihss_severe
- Mediator variable = sap7days
- List covariates = i.gender ----> i.onsetarrival_dys_cat
- Option a0() = referent exposure level, a1() actual exposure level
- Option m() = indicator of mediator presence
- yreg() = definition of outcome regression model, mreg() = definition of mediator regression model
- yregoptions()/mregoptions() = standard stata options for each regression model e.g. definition of estimation methods or output such as odds ratios, here includes clustering/shared frailty around the stoke units
- c() = covariate values, fixes values for each covariate inorder to compute 4-way decomposition here fixed at reference categories
- full = provides full output

Additional options used in this study but not included in the command above related to obtaining confidence intervals via bootstrapping. In this study a shared frailty model for the outcome was used to attempt to account for clustering in the stroke units. Given the significant sample size and number of stroke units present this required significant computational power. Due to limited time and resources, the additional bootstrapping was not viable. We chose to report the shared frailty models, due our preconceived theory that clustering was a more significant issue than departures from the parametric assumptions given the sample size present. Sensitivity analysis not reported here, with bootstrapping but no shared frailty indicated little difference to the confidence intervals and the conclusions reported.

Full description of the ‘med4way’ command, including additional post estimation commands, can be found in the paper (see link above), and Stata help menu.
